# Automated Classification of Significant Prostate Cancer on MRI: A Systematic Review on the Performance of Machine Learning Applications

**DOI:** 10.3390/cancers12061606

**Published:** 2020-06-17

**Authors:** Jose M. Castillo T., Muhammad Arif, Wiro J. Niessen, Ivo G. Schoots, Jifke F. Veenland

**Affiliations:** 1 Department of Radiology and Nuclear Medicine, Erasmus MC, 3015 GD Rotterdam, The Netherlands; a.muhammad@erasmusmc.nl (M.A.); w.niessen@erasmusmc.nl (W.J.N.); i.schoots@erasmusmc.nl (I.G.S.); j.veenland@erasmusmc.nl (J.F.V.); 2 Faculty of Applied Sciences, Delft University of Technology, 2600 AA Delft, The Netherlands; 3 Department of Radiology, Netherlands Cancer Institute, 1066 CX Amsterdam, The Netherlands; 4 Department of Medical Informatics, Erasmus MC, 3015 GD Rotterdam, The Netherlands

**Keywords:** prostate carcinoma, clinically significant, radiomics, machine learning, deep learning, systematic review, mpMRI, classification, model, prediction, Gleason score

## Abstract

Significant prostate carcinoma (sPCa) classification based on MRI using radiomics or deep learning approaches has gained much interest, due to the potential application in assisting in clinical decision-making. Objective: To systematically review the literature (i) to determine which algorithms are most frequently used for sPCa classification, (ii) to investigate whether there exists a relation between the performance and the method or the MRI sequences used, (iii) to assess what study design factors affect the performance on sPCa classification, and (iv) to research whether performance had been evaluated in a clinical setting Methods: The databases Embase and Ovid MEDLINE were searched for studies describing machine learning or deep learning classification methods discriminating between significant and nonsignificant PCa on multiparametric MRI that performed a valid validation procedure. Quality was assessed by the modified radiomics quality score. We computed the median area under the receiver operating curve (AUC) from overall methods and the interquartile range. Results: From 2846 potentially relevant publications, 27 were included. The most frequent algorithms used in the literature for PCa classification are logistic regression (22%) and convolutional neural networks (CNNs) (22%). The median AUC was 0.79 (interquartile range: 0.77–0.87). No significant effect of number of included patients, image sequences, or reference standard on the reported performance was found. Three studies described an external validation and none of the papers described a validation in a prospective clinical trial. Conclusions: To unlock the promising potential of machine and deep learning approaches, validation studies and clinical prospective studies should be performed with an established protocol to assess the added value in decision-making.

## 1. Introduction

Prostate cancer (PCa) is the most common malignancy and second leading cause of cancer-related death in men [1]. One in six men will develop PCa; however, many pathological subclassifications are present, roughly separated into low grade and high grade, or into clinically insignificant and significant PCa. Significant PCa (sPCa) has the potential to metastasize and therefore has the poorest prognosis. Insignificant PCa may not metastasize and mostly results in indolent or slowly growing low-grade tumors. Patients with low-grade PCa die from other causes than PCa. Overdiagnosis of low-grade PCa and, consequently, overtreatment is an important problem in current practice, estimated to be up to 50% of all PCa [2]. Therefore, accurate discrimination between significant and low-grade PCa is critical for risk stratification and clinical decision-making

Multiparametric magnetic resonance imaging (mpMRI) has become an essential tool for PCa risk assessment. This is performed by radiologists using the Prostate Imaging Reporting and Data System (PI-RADS) [3]. However, mpMRI assessment is challenging and prone to inter- and intrareader variability, making this evaluation dependent on reader experience [4].

Quantitative assessment of mpMRI might provide a radiologist with an objective and noninvasive tool to support decision-making in clinical practice and decrease the intra- and inter-reader discordances. Due to the increased interest in AI applications in medicine, increased computer power, and the development of new AI techniques, the number of studies proposing computer-aided diagnosis (CAD) systems to detect and classify tumors on medical images using either radiomics and machine learning (ML) or deep learning (DL) methods has greatly expanded. This certainly is the case for PCa detection and classification. Whereas the frontrunners focused primarily on the proof of concept of using radiomics and machine learning techniques to classify prostate tumor versus no tumor or versus benign lesions, the more recent literature focuses on the clinically relevant problem of discriminating significant from low-grade tumors. In older studies, the Gleason grades of the included tumors are often not mentioned, making it difficult to compare results between studies.

In recent literature, a large variety of classifiers have been applied based on different ground truths (biopsies or prostatectomy data) and using several performance statistics, with varying results. Our aim with this study was to systematically review the literature to synthesize and describe the state of the art and current practice for automated significant PCa classification based on mpMRI. Therefore, we identified the following questions: (1) What algorithms are most frequently used for sPCa classification? (2) Is there a relation between the performance and the method or MRI sequences used? (3) Which study design factors affect the performance on sPCa classification? (4) Have methods been validated in a clinical setting?

## 2. Results

### 2.1. Search Results and Statistical Description

**Figure 1:**
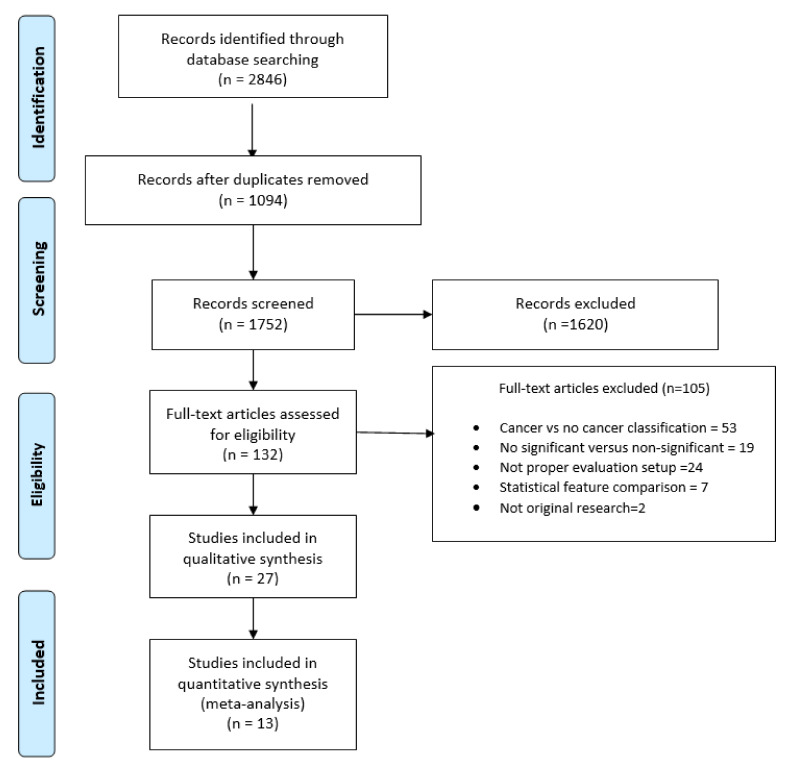
Systematic review flow diagram, modified from [32].

The flow diagram is depicted in Figure 1. In total, 27 articles were eligible for inclusion in this review [5,6,7,8,9,10,11,12,13,14,15,16,17,18,19,20,21,22,23,24,25,26,27,28,29,30,31]. From these, 13 studies reported enough information to perform a meta-analysis.

**Figure 2:**
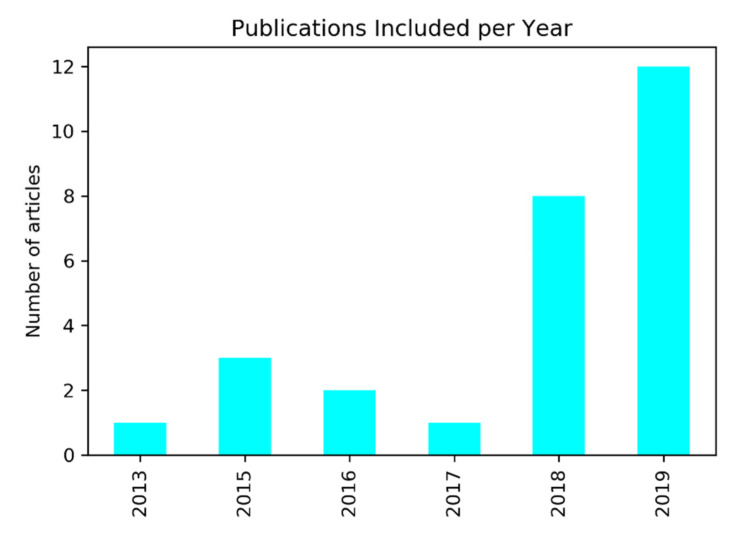
Number of articles included per year of publication.

In Figure 2, the number of publications per year included in our analysis is shown. The first study dates from 2013. Most studies included in the systematic review were published in the years 2018 and 2019. The studies included obtained a median score of 52% on the modified radiomics quality score with an interquartile range (IQR) = 44–56%.

Regarding the study design, in five studies (17%), the data acquisition had a prospective design, while 22 (83%) studies used a retrospective design for data gathering. In all studies, the computer-aided analysis (CAD) was performed retrospectively. The median of the dataset size was 127 patients with an IQR of 71–193 patients, the largest dataset had 344 patients, and the smallest counted 36 patients. Most of the data were collected internally in a single-center setting (66%); a minor proportion (30%) used only publicly available datasets. Furthermore, only one study used data from several centers (4%) (Table S1).

When observing the population description used for PCa classification, 89% described the Gleason distribution of their study population. Almost 90% of the studies described their study population in detail. Seven studies (29%) mentioned a size restriction for the inclusion of lesions.

As input for the classification method, the Apparent diffusion coefficient (ADC) map was the most frequently used (93%), followed by the T2w sequence (81%). Dynamic contrast-enhanced (DCE) images were used in 52% of the studies. Most studies used more than two image inputs for the classification method. Twenty-two papers gave a description of the MRI protocols used. Twenty-four of the studies (89%) used data acquired with a 3T MRI. Given that 25 of the studies were performed with a single-center cohort, most of the datasets originated from a single scanner or several scanners belonging to the same vendor. Only three studies used an external validation cohort to check the generalizability of their results (Table S2).

For classification, eight studies (30%) used prostatectomy as a reference standard. The PCa lesion correlation with the mpMRI and the delineation were mostly carried out manually by an experienced radiologist (70%), while fully automatic and semiautomatic methods were rarely applied (7%), and other studies did not comment on how the segmentation was obtained (23%).

From the image combination selected by the researchers, image feature extraction was performed. The variation in number of features selected for the development of the model was large: median = 8 features and IQR of 3–56 features. 

The classification task most observed was the discrimination between PCa tumors with GS ≥ 7 and GS ≤ 6 (62%). Other classification tasks performed were ISUP classification, differentiation between Gleason pattern 3 + 4 and Gleason 4 + 3, single Gleason pattern 4 vs single Gleason pattern 3, and prediction. Additionally, these classification tasks were performed without distinction of the tumor location in 17 studies (63%), while 4 studies (15%) reported the results per zone. Four studies focused on tumors in the peripheral zone (PZ) (15%).

For the classification based on the imaging features, several ML algorithms were used, as can be seen in Table 1. Logistic regression, convolutional neural networks, and random forest were most frequently chosen, while support vector machines (SVMs) and the linear mix model were selected slightly less frequently. The rest of the ML algorithms were not reported more than twice (Table S3).

All studies performed a thorough cross-validation. Eleven studies (41%) reported a class imbalance between the sPCa and non-sPCa cases, and performed an upsampling of the minority class. Eight of these studies (30%) reported the use of a data augmentation method. Three of these papers also performed data augmentation on the validation set. In 24 studies (89%), the validation of the methods was performed with an internal dataset, while only 3 studies used external data to check the generalizability of their results. Only in one study was the performance of a radiologist with or without CAD compared [24].

The performance of the methods was reported by the authors in terms of the area under the receiver operating curve (AUC), accuracy, sensitivity, and/or specificity. However, not all the metrics were reported all the time, and only 59% of the included studies reported confidence intervals. For instance, 41% of the studies did not report metrics as sensitivity and specificity. Only 33% reported their metrics per zone, and most studies reported their metrics for the whole prostate.

The median AUC of all the studies included was 0.79 (IQR: 0.77–0.87). 

### 2.2. Factors Influencing the Performance

In Table 1, the performance of the different methods is summarized. As can be seen, the support vector machine method, the linear mix model, and k-nearest neighbor occupy the top three positions in terms of performance. The methods validated on external datasets are marked with an (*).

**Figure 3:**
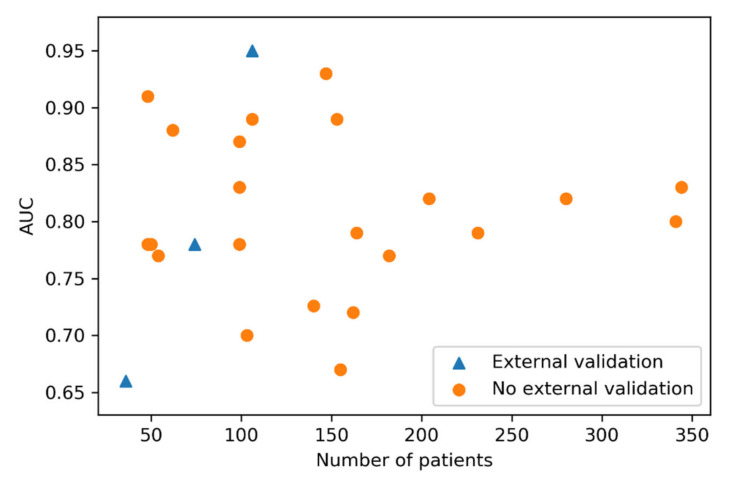
Scatter plot showing number of patients included per study and the performance reported. The studies with a blue triangle were externally validated and studies marked with an orange circle were tested internally.

As demonstrated in Figure 3, the reported performances do not show a correlation with the number of patients included in the study. Furthermore, we found no significant statistical difference between the performance of externally validated studies and studies without external validation (Mann–Whitney *U* test = 43, *p*-value = 0.49). 

**Figure 4:**
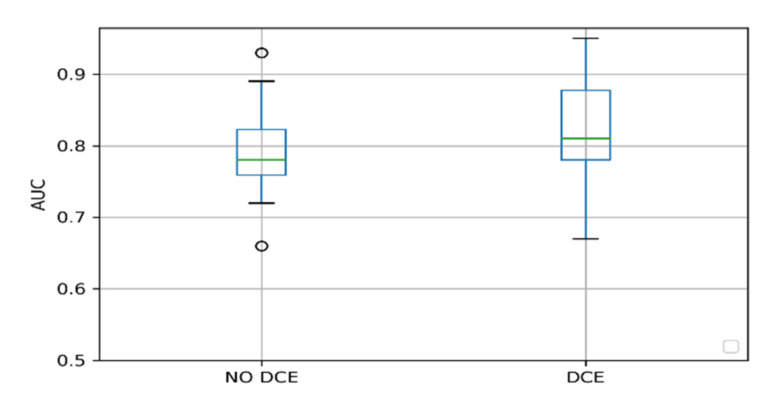
Model performance boxplots grouped by inclusion of the dynamic contrast-enhanced (DCE) sequence for model development.

We also investigated whether the inclusion of the DCE sequence for model development influenced the reported performance of the model. As can be seen in Figure 4, the AUC of models including DCE overlaps with the IQR of the models not using the DCE sequence. There was no statistically significant difference between the median AUC between the two groups (Mann–Whitney *U* test = 61.0, *p*-value = 0.12). 

**Figure 5:**
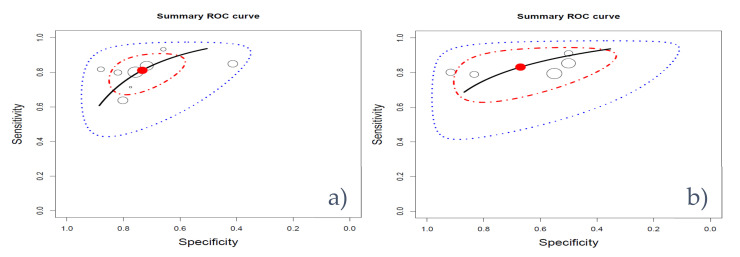
Hierarchical receiver operating characteristic curves for biopsies (**a**) and prostatectomies (**b**). Studies are represented by circles with a size proportional to their data size, red = optimal working point, black line = curve, red line = 95% confidence region, blue line = 95% predicted region.

Similarly, we tested if there were differences in performance between studies using transrectal ultrasound (TRUS) guided biopsies or prostatectomies as the reference standard. We found no statistical difference between the median AUC of models using TRUS-guided biopsies and prostatectomies as the reference standard (Mann–Whitney *U* test = 56.5, *p*-value = 0.2). Additionally, for the studies that reported sensitivity and specificity, we computed the hierarchical receiver operating characteristic curves (Figure 5). The studies using biopsies showed more homogenous performance compared with the ones using prostatectomy. Furthermore, the difference between the confidence regions and predicted region was less prominent.

## 3. Discussion

In this study, we systematically reviewed the literature and described the state of the art and current practices for significant PCa classification based on multiparametric MRI. In recent years, many studies have been published using radiomics in combination with machine learning or deep learning methods on mpMRI to classify significant prostate cancer with the ultimate goal of assisting the radiologist in his diagnosis workflow. In this systematic review, we quantified the chosen approaches for radiomics, and deep learning methods applied and summarized the results for PCa classification. Despite the promising results obtained by several studies and their explicit intention to translate their tools into clinics, none of the studies demonstrated the improvement of PCa detection using a CAD system in a clinical workflow.

This lack of prospective studies might be explained by several reasons: First, performing a prospective study embedded in the clinical workflow is more time and cost intensive per patient than performing a retrospective study. Second, a large cohort of patients will be needed to demonstrate with enough power the added value of a CAD system. In most studies, the methods are trained and validated on MRI data from the full spectrum of ISUP classes, whereas the radiologist primarily needs assistance for the ISUP classes 1–3 [33]. Further, tumors with a larger volume are easier to detect. Zhu et al. [24] found that for lesions larger than 1.5 cm, the CAD system did not improve the sensitivity. Most studies described the distribution of Gleason grades in their study but failed to give a distribution of the sizes of the tumors. Both aspects can have a major impact on the performance of the CAD. For a prospective study to assess the improved performance of the radiologist working with the CAD system, the cohort should be large enough to contain enough intermediate GS and tumors with varied sizes.

With this perspective, most of the studies are proof-of-concept studies. Researchers tend to work with data that are accessible and less time consuming to obtain, which would explain the observation of larger patient cohorts per study when using TRUS-guided biopsies as the reference standard as compared with patient cohorts in studies that used prostatectomies. In fact, we need patient cohorts with prostate template mapping biopsies (at 5 mm intervals) for appropriate diagnostic test accuracies, as well as second best cohorts with TRUS-guided biopsies combined with MRI-targeted biopsies (in MRI-positive cases). Although radical prostatectomy specimen may be the best reference standard, we need to be aware that this is already a selected patient cohort, with most likely high(er) risks and high(er) Gleason grade PCa, which is not representative of the screened or tested population, in which CAD could be helpful. Men on active surveillance are excluded in such high-risk cohorts.

Similarly, the time and cost limitations also apply for MRI data, where obtaining multicentric datasets for radiomics studies is an obstacle to overcome [34]. In the context of PCa, this means gathering data from urology, radiology, and pathology departments. Data must be anonymized, processed, matched, segmented, and verified before being used for CAD development. Therefore, it is not surprising that the images used by most of the studies included in this systematic review were generated using a single scanner or two scanners of the same vendor in one center. This should be considered as limiting when aiming to develop a generalizable model. Due to the feature dependency on the acquisition parameters of scanners [35], for developing a generally applicable model, data from different scanners and different sites will be needed.

We found no performance difference for studies that included T2, diffusion-weighted imaging (DWI), and ADC images as compared to studies that added a DCE sequence [36]. The DCE sequence is included in the PIRADS v2; however, there is a debate about the added value. Based on the results of this systematic review, adding the DCE sequence in a proposed study cohort may not enhance the performance of the methods significantly. A more in-depth analysis to investigate the added value of DCE in particular circumstances is needed.

A significant number of the studies included in this systematic review specified their patient inclusion/exclusion criteria, describing how large the patient cohort was and how many tumor samples were taken to develop the model. Nevertheless, the details regarding the selected populations were heterogenous between studies. Some papers limited their description to the number of positive and negative samples [27,31], while others mentioned the PCa lesion distribution per GS [11,23] and/or the volume distribution [16,21]. Both characteristics describe the type of population used and whether the PCa lesions are clinically relevant [2]. Moreover, this information is fundamental when drawing conclusions regarding the added value in a clinical setting of the proposed method. For instance, if a model is trained exclusively on tumors with a first Gleason pattern > 4 and a volume > 5 mL, the added value in a clinical setting would be zero, since these lesions will be easily detected not only on MRI by a radiologist but also on rectal examination by a urologist. A model trained on small tumors with volumes between 0.5 and 1 mL and with the aim to differentiate between GS 3 + 3 (ISUP grade 1) and 4 + 3 (ISUP grade 3) might represent a significant support tool. As previous investigations have suggested that GS 7 tumor volumes above 0.5 mL and GS6 tumor volumes above 1.3 mL become clinically significant, we may consider thresholding CAD systems to such tumor volumes [37].

Regarding the correlation of the tumors on MRI with the pathology reference, the most common practice is to perform a segmentation by an expert radiologist. The region of interest (ROI) delineation is a factor that has a direct influence on the feature computation [38]. Therefore, studying the robustness of the features to the segmentation is a factor that authors should consider when validating their methods. This process can also by automated by performing both segmentation and classification within the same pipeline. Nevertheless, the performance of segmentation methods was outside the scope of this review.

To enable a fair comparison of the different methods, only studies that compared the classification of significant versus nonsignificant tumors were included. Studies that discriminated healthy tissue from ISUP 1–5 were not included. Furthermore, to enhance comparability, only studies that performed the evaluation study on a patient or lesion level were included. A number of studies performed the evaluation on the voxel, slice, or segment level, reporting excellent AUCs. However, this does not correspond to the output that a radiologist will use in a clinical context. Furthermore, some studies did not perform a patient split for the training and validation cohort, which may lead to overestimated results [39]. Therefore, these studies were also excluded. Of the included studies, 11 reported class imbalance and used resampling and data augmentation techniques to balance the classes. However, three studies also performed data augmentation on the validation set, which may make the results overoptimistic.

When the features computed from the ROI are used to develop a classification model, the number of features is most likely to be higher than the number of samples included in the study, which increases the risks of obtaining an overfitted model. As a consequence, authors aim to reduce the feature space by removing the less informative and redundant features. However, when reporting their results, the number of features selected is vague in most of the cases. Furthermore, most researchers do not offer details regarding preprocessing steps and model parameters. Only a few authors made their code publicly available in a repository [40]. This lack of detailed information hampers the reproducibility of the results, makes it difficult to compare methods, and does not help in pushing this field forward. 

Most of the papers described a single-center study without external validation. Exchanging codes and data between different research groups would help to externally validate the different methods and to improve the robustness of the methods for different centers and scanners. Moreover, only one study compared the performance of radiologists with or without a CAD. As the ultimate goal of these approaches is to assist the radiologist in this diagnostic task, the real evaluation should be performed in a clinical setting

The performance obtained by ML and DL methods included in this systematic review can be considered comparable. While the SVM, the linear mix model, and the k-nearest neighbor showed the highest performance, most studies were not validated with an external set, so the reported performance will most likely be overestimated. On the other hand, for linear mixed models and random forests, there is scientific evidence that their real performance is stable when translating these methods to a new population with similar characteristics as the population used to create the model [41].

All of the included studies, except for one, focused on the imaging data, whereas in urology, the use of decision models based on clinical data is common practice. We expect that the combination of imaging and clinical data in a more patient-based model will further improve the performance 

The literature suggests that there are discordances between GS in TRUS biopsies and the GS obtained after prostatectomy [42], where the latter is more accurate since the whole organ is assessed and there are no sampling errors. We hypothesized that the classification performance obtained while developing a model using prostatectomies as the reference standard would obtain better results when compared with using TRUS-guided biopsies. However, our results suggest that an equivalent performance can be obtained, regardless of the reference being used. This could be explained by the fact that using biopsies allows a larger patient cohort in the study, which might mitigate the effects of sampling errors and generalize as well as a study with less patient samples based on prostatectomies.

In a broader perspective, there are many factors to consider when developing a PCa classification model. In the previous paragraphs, we described how these factors might influence the end model performance and generalizability. However, the current heterogeneity regarding how these relevant factors are described in studies makes it hard to make a fair comparison between them. Therefore, we would like to make some recommendations that are fundamental from our perspective when working on PCa classification: (1) to use a prospective study design to assess the added value in a clinical setting with the inclusion of clinical parameters in the model and with a clear description of the patient cohort, the inclusion/exclusion criteria, the risk group, patient age, number of lesions included per patient, and the distribution according to GS and tumor volume; (2) to encourage the sharing of codes and data; (3) to test the model on external datasets; and (4) to report the model and the performance metrics in a standardized way. 

This study has some limitations that should be mentioned. First, due to publication bias, methods with low performances were not included in this study [32]. As a result, the real performance of the listed methods in this systematic review might be overestimated. Second, some papers might have been excluded from this systematic review since the necessary information to assess the eligibility could not be obtained from the test. Finally, only 12 papers (43%) from the 29 papers included in the systematic review reported sufficient information to perform a meta-analysis, which means that our conclusions in this section were based on less than half of the final number of papers included.

## 4. Materials and Methods 

This systematic review was conducted following the recommendations published in the Preferred Reporting Items for Systematic Reviews and Meta-Analyses for Diagnostic Test Accuracy (PRISMA-DTA) statement [43]. A systematic literature search was performed on 6 January 2020 on the online databases EMBASE and MEDLINE Ovid. The databases were searched for primary publications describing studies of classification and/or detection of clinically significant PCa on MRI employing radiomics combined with a machine learning and/or a deep learning approach. The exact search can be found in the Supplementary Materials Section S1.

After removal of duplicate findings, the screening of abstracts and full-text articles was performed independently by two researchers (J.M.C.T. and J.F.V.). Discrepancies were resolved by discussion.

The following exclusion criteria were used: not original research, studies that performed PCa classification without description of Gleason grades of the tumors, studies that did not classify significant versus nonsignificant PCa, studies that did not have a proper evaluation setup, and studies that performed only a statistical feature comparison. For the data extraction, three researchers performed a training phase (J.M.C.T., M.A., and J.F.V.), where they discussed the data extraction from 4 randomly selected articles to check criteria agreement on the items of the predefined extraction form. Following this training phase, these authors independently extracted the items. The data extraction was cross-checked. When a study reported several classification experiments or compared several feature classifier combinations, the best performance results were extracted. Missing specificity values were computed from the sensitivity, positive predictive value, and patient numbers. When authors reported the performance metrics for 5 separate ISUP classes, the performance metrics for class 1 versus classes 2–5 were computed. When performance metrics were reported per zone, the average was computed for the whole prostate. When performance metrics for an augmented and a nonaugmented validation set were reported, the metrics from the nonaugmented set were extracted.

To assess the quality of the included studies, we defined our own system for PCa classification study quality assessment. Our assessment tool was based on the Radiomics Quality Score (RQS) [44]. This tool has been developed for radiomics studies; several items are applicable not only for radiomics studies but also for other ML approaches. Nevertheless, the RQS is quite extensive and does not include criteria to evaluate biases regarding patient inclusion criteria, which in our opinion is a fundamental point to assess in classification studies. Therefore, we included in our assessment tool this criterion taken from the Quality Assessment of Diagnostic Accuracy Studies (QUADAS) [45]. The selected criteria for our quality assessment are included in Table 2.

After performing a qualitative analysis on the included papers, we performed a statistical analysis on several factors that could influence the performance matrix. We performed a meta-analysis with the studies that contained the detailed information required: area under the curve, sensitivity, and specificity for the significant versus nonsignificant PCa classification. Statistical analysis and meta-analysis were performed with two free source programing languages: Python and R, respectively. The hierarchical receiver operating curves were computed using the software package HSROC in R [46]. For statistics computation regarding studies with multiple performance outcomes, we used the highest performance metric reported.

## 5. Conclusions

This systematic review shows an increased research interest in PCa classification with machine learning and deep learning approaches, and many promising results are reported. Among such studies, large heterogeneity is present in patient population (and risks), number of included patients and prostate cancer lesions, MRI sequences used, machine learning and deep learning approaches, and used reference standards. No significant differences were identified in diagnostic performance regarding these factors.

This review also shows that external validation of methods is lacking, and that there are no studies describing the actual inclusion of radiomics or deep learning models in a clinical workflow. To unlock the promising potential of machine and deep learning approaches, validation and prospective studies should be performed in a clinical setting with an established protocol to assess the added value in decision-making. 

## Figures and Tables

**Table 1:** Method performances in terms of the median area under the receiver operating curve (AUC) and respective interquartile range (IQR).

Method	Median (AUC)	IQR		N
SVM	0.91	0.84	0.92	3
Linear mix model *	0.89	0.84	0.92	3
k-nearest neighbor	0.87	0.87	0.87	1
Neural Network	0.81	0.76	0.84	2
CNN	0.80	0.78	0.83	6
Random Forest *	0.80	0.75	0.82	4
Logistic regression	0.79	0.77	0.79	6
Linear discriminant analysis	0.74	0.72	0.76	2

(*) Models validated with an external dataset. N = number of studies using the method. CNN = convolutional neural network and SVM = support vector machine.

**Table 2:** Prostate Carcinoma Classification Study Quality Score, modified from radiomics quality score [44].

Criteria	Points	Min	Max
Bias due to population selection	Patient selection introduces bias (−5), patient selection might introduce bias 0, patient selection does not introduce bias (+5).	−5	5
MRI protocol description	MRI protocols are well documented (+1).	0	1
Multiple segmentations	Study includes segmentations from several physicians/algorithms/software. Study analyzes method robustness to segmentation variabilities (+1).	0	1
Multivariable analysis with non-radiomics features	Study includes multivariable analysis with non-radiomics features, for instance, age and prostate-specific antigen (+1).	0	1
Discrimination statistics	The study reports discrimination statistics (for example, receiver operating curve, AUC) (+1). The study reports the validation method (for example, cross-validation, bootstrapping) and confidence intervals (+1).	0	2
Prospective study	The study has a prospective design (+7).	0	7
Validation	The study does not report a validation method (−5), a validation performed on a dataset from the same institute (+2), a validation on a dataset from another institute (+3), a validation on a dataset from two distinct institutes (+4), a validation on a dataset from three different institutes (+5). *Validation set size should be of comparable size and representative of the training set.	−5	5
Comparison to gold standard	The study assesses the extent to which the model agrees with/is superior to the current “gold standard” (+2).	0	2
Report potential clinical utility	The study reports potential clinical utility and potential application of the model in a clinical setting (+2).	0	2
Open science and data	Study scans are open source (+1), region of interest (ROI) segmentations are open source (+1). The classification model with parameter settings is publicly available (+1).	0	3
Score	(number of points × 100)/29	0%	100%

## Supplementary Materials

The following are available online at https://www.mdpi.com/2072-6694/12/6/1606/s1, Supplementary Materials Section S1: Search strategy, Table S1: Table with summary of extracted parameters regarding data sets from included papers, Table S2: Table with summary of extracted parameters regarding MRI data from included papers, Table S3: Table with summary of extracted parameters regarding model’s performance and methodology from included papers.
