# Supplementary material for: Automated Classification of Significant Prostate Cancer on MRI: A Systematic Review on the Performance of Machine Learning Applications
Source: Cancers (Basel). 2020 Jun 17;12(6):1606. doi: 10.3390/cancers12061606 (PMC7352160; doi:10.3390/cancers12061606)
Supplement: Supplementary file 1 [file cancers-12-01606-s001.pdf]

Supplementary materials

Automated Classification of Significant Prostate Cancer on MRI: A Systematic Review of the Performance of Machine Learning Applications

Jose M. Castillo T., Muhammad Arif, Wiro J. Niessen, Ivo G. Schoots and Jifke F. Veenland

Supplementary Materials Section S1: Search strategy.

The searches consisted of the following keywords combination:

Embase.com: ('prostatic neoplasms'/exp OR (prosta* AND (neoplas* OR cancer* OR tumour* OR tumor* OR carcinoma*):ab,ti)) AND ('magnetic resonance imaging'/exp OR (MRI* OR 'magnetic resonance' OR 'mr imaging'):ab,ti) AND (characteri* OR agressiven* OR differentiat* OR accur* or distinguish* OR gradi* OR classif* OR stratif* OR staging* OR diagnos* OR identi* OR predict* OR detect* OR discrimin*):ab,ti AND ('machine learning'/exp OR (model* OR (machine AND learning) OR CNN OR neural OR computer* OR automat*) :ab,ti) NOT (conference abstract)/lim

MEDLINE ovid: ("Prostatic Neoplasms"/ OR (prostat* AND (neoplas* OR cancer* OR tumour* OR tumor* OR carcinoma*)).ab,ti,kf.) AND ("Magnetic Resonance Imaging"/ OR (MRI* OR "magnetic resonance" OR " mr imaging").ab,ti,kf.) AND ((characteri* OR agressiven* OR differentiat* OR accur* or distinguish* OR gradi* OR classif* OR stratif* OR staging* OR diagnos* OR identi* OR predict* OR detect* OR discrimin*).ab,ti,kf.) AND ("machine learning"/ OR (model* OR (machine AND learning) OR CNN OR neural OR computer* OR automat*).ab,ti,kf.)

Table S1. Table with summary of extracted parameters regarding data sets from included papers.

Firs Author Name	Year	Type of Study	Population Description Present	Data Origin	Number of Patients	Number of Lesions	Classification Problem	Ground Truth	Positive Class Proportion	Gleason Distribution Described
**Abraham B. [5]**	2018	Retrospective	yes	Public	162	182	ISUP 5 classes	Biopsies	40	yes
**Abraham B. [6]**	2019	Retrospective	yes	Public	??	100	ISUP 5 classes	Biopsies	68	yes
**Antonelli M. [17]**	2019	Prospective	yes	Local	164	164	GS 4 vs GS 3	Biopsies	63	yes
**Chaddad A. [25]**	2018	Retrospective	yes	Public	99	99	GS 6 vs 7(3 + 4) vs ≥ 7 (4 + 3)	Biopsies	69	yes
**Chaddad A. [26]**	2018	Retrospective	yes	Public	99	99	GS ≤ 6 /7(3 + 4)/≥ 7(4 + 3)	Biopsies	69	yes
**Chen Q. [27]**	2019	Retrospective	yes	Public	344	538	GS ≥ 7 vs ≤ 6	Biopsies	23	yes
**Chen T. [28]**	2018	Retrospective	yes	Local	182	184	GS ≥ 7 vs ≤ 6	Biopsies	78	yes
**Dikaios N. [30]**	2015	Retrospective	yes	Local	155	0	GS ≥ 7 vs ≤ 6	Biopsies	40	No
**Dikaios N. [29]**	2015	Retrospective	yes	Local	231	0	GS ≥ 7 vs ≤ 6	Biopsies	38	yes
**Dinh A.H. [31]**	2018	Prospective	yes	Local	106	104	GS ≥ 7 vs ≤ 6	Biopsies	42	yes
**Dinh A.H. [7]**	2016	Prospective	yes	Local	106	104	GS ≥ 7 vs ≤ 6	Prostatectomy	83	yes
**Fehr D. [8]**	2015	Retrospective	yes	Local	147	193	GS ≥ 7 vs ≤ 6	Prostatectomy	82	yes
**Jensen C. [9]**	2019	Retrospective	yes	Public	99	112	ISUP 5 classes	Biopsies	68	yes
**Kwon D. [10]**	2018	Retrospective	yes	Public	204	330	GS ≥ 7 vs ≤ 6	Biopsies	70	No
**Li J. [11]**	2018	Retrospective	yes	Local	48	152	GS ≥ 7 vs ≤ 6	Biopsies	83	yes
**Li Q. [12]**	2019	Retrospective	yes	Local	103	167	GS ≥ 7 vs ≤ 6	Biopsies	27	yes
**Maas M.C. [13]**	2019	Prospective	yes	Local	50	65	GS ≥ 7 vs ≤ 6	Prostatectomy	57	yes
**Mehrtash A. [14]**	2017	Retrospective	yes	Public	341	529	GS ≥ 7 vs ≤ 6	Biopsies	23	yes
**Peng Y. [15]**	2013	Retrospective	yes	Local	48	61	Low Grade (3 + 4) and normal tissue vs cancer tissue	Prostatectomy	77	yes
**Penzias G. [16]**	2018	Retrospective	yes	Local + External	36	105	GS ≥ 7 vs ≤ 6	Prostatectomy	55	yes
**Rozenberg R. [18]**	2016	Retrospective	yes	Local	54	54	GS 3 + 4 vs 4 + 3	Prostatectomy	26	No
**Schelb [19]**	2019	Retrospective	yes	Local	312	457	GS ≥ 7 vs ≤ 6	Biopsies	38	yes
**Toivonen J. [20]**	2019	Prospective	yes	Local	62	100	GS 3 + 3 vs >6	Biopsies	80	yes
**Transin S. [21]**	2019	Retrospective	yes	Local	74	127	GS ≥ 7 vs ≤ 6	Prostatectomy	70	yes
**Min X. [22]**	2019	Retrospective	No	Local	280	280	GS ≥ 7 vs ≤ 6	Biopsies	86	yes
**Zhong X. [23]**	2019	Retrospective	yes	Local	140	216	GS ≥ 7 vs ≤ 6	Prostatectomy	49	yes
**Zhu L. [24]**	2019	Retrospective	yes	Local	153	116	GS ≥ 7 vs ≤ 6	Biopsies	64	yes

GS: Gleason score.

Table S2. Table with summary of extracted parameters regarding MRI data from included papers.

First Author Name	Year	Protocol Present	Vendor 1	Scanner	M. F (T)	Vendor 2	Scanner 2	M. F (T)	T2	ADC	DWI	DCE
**Abraham B. [5]**	2018	No	Siemens	Magnetom	3	Siemens	Skyra	3	Yes	Yes	Yes	No
**Abraham B. [6]**	2019	No	Siemens	Trio-trim	3	-	-	-	Yes	Yes	Yes	No
**Antonelli M. [17]**	2019	Yes	Philips	Achieva	3	-	-	-	Yes	Yes	No	Yes
**Chaddad A. [25]**	2018	No	Siemens	Magnetom	3	Siemens	Skyra	3	Yes	Yes	No	No
**Chaddad A. [26]**	2018	Yes	Siemens	Magnetom	3	-	-	-	Yes	Yes	No	No
**Chen Q. [27]**	2019	Yes	Siemens	Magnetom Trio	3	Siemens	Skyra	3	Yes	Yes	No	Yes
**Chen T. [28]**	2018	Yes	Philips	Intera Achieva	3	-	-	-	Yes	Yes	Yes	No
**Dikaios N. [30]**	2015	Yes	Siemens	Avanto	1.5	-	-	-	Yes	Yes	No	Yes
**Dikaios N. [29]**	2015	Yes	-	-	1.5	-	-	-	Yes	Yes	No	No
**Dinh A.H. [31]**	2018	Yes	GE	-	3	Philips	Achieva	3	No	Yes	No	No
**Dinh A.H. [7]**	2016	No	-	-	-	-	-	-	Yes	Yes	No	Yes
**Fehr D. [8]**	2015	Yes	GE	Signa HDX	3	-	-	-	Yes	Yes	No	No
**Jensen C. [9]**	2019	Yes	Siemens	Trio-trim	3	Siemens	Skyra	3	Yes	Yes	Yes	Yes
**Kwon D. [10]**	2018	Yes	Siemens	Trio-trim	3	Siemens	-	3	Yes	Yes	Yes	Yes
**Li J. [11]**	2018	Yes	Philips	Achieva	3	-	-	-	No	Yes	No	Yes
**Li Q. [12]**	2019	No	Siemens	-	1.5	-	-	-	Yes	Yes	No	Yes
**Maas M.C. [13]**	2019	Yes	Siemens	Magnetom Skyra	3	-	-	-	Yes	Yes	Yes	Yes
**Mehrtash A. [14]**	2017	Yes	Siemens	Trio-trim	3	-	-	-	No	Yes	Yes	Yes
**Peng Y. [15]**	2013	Yes	Philips	Achieva	3	-	-	-	Yes	Yes	No	No
**Penzias G. [16]**	2018	Yes	Siemens	Verio	3	Philips	-	3	Yes	No	No	No
**Rozenberg R. [18]**	2016	Yes	Siemens	Magnetom	3	-	-	-	No	Yes	No	No
**Schelb [19]**	2019	Yes	Siemens	Prisma	3	-	-	-	Yes	No	Yes	No
**Toivonen J. [20]**	2019	Yes	Philips	Ingenuity	3	-	-	-	Yes	Yes	No	Yes
**Transin S. [21]**	2019	Yes	Siemens	Trio-trim	3	-	-	-	No	Yes	No	Yes
**Min X. [22]**	2019	Yes	Siemens	-	3	-	-	-	Yes	Yes	Yes	No
**Zhong X. [23]**	2019	Yes	Siemens	Trio-trim	3	-	-	-	Yes	Yes	No	No
**Zhu L. [24]**	2019	Yes	Philips	Achieva	3	-	-	-	Yes	Yes	Yes	No

M.F: Magnetic field in Tesla.

Table S3. Table with summary of extracted parameters regarding model's performance and methodology from included papers.

Firs Author Name	Year	Method	Balancing of Data	Data Augmentation	AUC	Sensitivity	Specificity	External Validation	Validation Radiologist using CAD
**Abraham B. [5]**	2018	Neural Network	No	No	0.72	0.8	0.55	No	No
**Abraham B. [6]**	2019	CNN+ ordinal class classifier	Yes	No	0.78	0.91	0.5	No	No
**Antonelli M. [17]**	2019	Logistic regression	Yes	Yes	0.79	0.85	0.5	No	No
**Chaddad A. [25]**	2018	Random Forest	Yes	No	0.83	-	-	No	No
**Chaddad A. [26]**	2018	Random Forest	Yes	No	0.78	-	-	No	No
**Chen Q. [27]**	2019	CNN	Yes	Yes	0.83	-	-	No	No
**Chen T. [28]**	2018	Logistic regression	Yes	Yes	0.77	0.83	0.67	No	No
**Dikaios N. [30]**	2015	Logistic regression	No	No	0.67	-	-	No	No
**Dikaios N. [29]**	2015	Logistic regression	No	No	0.79	-	-	No	No
**Dinh A.H. [31]**	2018	Linear mix model	No	No	0.95	-	-	Yes	No
**Dinh A.H. [7]**	2016	Linear mix model	No	No	0.89	0.79	0.84	No	No
**Fehr D. [8]**	2015	SVM	Yes	Yes	0.93	0	0	No	No
**Jensen C. [9]**	2019	k-nearest neighbor	No	No	0.87	0.8	0.91	No	No
**Kwon D. [10]**	2018	Random Forest	No	No	0.82	0.8	0.76	No	No
**Li J. [11]**	2018	SVM	Yes	Yes	0.91	0.82	0.87	No	No
**Li Q. [12]**	2019	Linear discriminant analysis	No	No	0.70	0.79	0.82	No	No
**Maas M.C. [13]**	2019	Logistic regression	No	No	0.78	-	-	No	No
**Mehrtash A. [14]**	2017	CNN	Yes	Yes	0.8	-	-	No	No
**Peng Y. [15]**	2013	Linear discriminant analysis	No	No	0.78	-	-	No	No
**Penzias G. [16]**	2018	Random Forest	No	No	0.66	-	-	Yes	No
**Rozenberg R. [18]**	2016	SVM	No	No	0.77	0.71	0.78	No	No
**Schelb [19]**	2019	CNN	No	No	-	0.92	0.47	No	No
**Toivonen J. [20]**	2019	CNN	No	No	0.88	-	-	No	No
**Transin S. [21]**	2019	Linear mix model	No	No	0.78	0.85	0.42	Yes	No
**Min X. [22]**	2019	Logistic regression	Yes	Yes	0.82	0.84	0.72	No	No
**Zhong X. [23]**	2019	CNN	Yes	Yes	0.726	0.636	0.8	No	No
**Zhu L. [24]**	2019	Neural Network + radiologist	No	No	0.89	0.93	0.66	No	Yes

(−) Missing values in the manuscripts. CAD: computer aided diagnostic system.

© 2020 by the authors. Licensee MDPI, Basel, Switzerland. This article is an open access article distributed under the terms and conditions of the Creative Commons Attribution (CC BY) license (http://creativecommons.org/licenses/by/4.0/).
